# Protective Effect of Surfactant Inhalation against Warm Ischemic Injury in an Isolated Rat Lung Ventilation Model

**DOI:** 10.1371/journal.pone.0072574

**Published:** 2013-08-29

**Authors:** Akihiro Ohsumi, Fengshi Chen, Jin Sakamoto, Daisuke Nakajima, Masashi Kobayashi, Toru Bando, Hiroshi Date

**Affiliations:** Department of Thoracic Surgery, Graduate School of Medicine, Kyoto University, Kyoto, Japan; University of Pecs Medical School, Hungary

## Abstract

Warm ischemia-reperfusion injury remains a crucial issue in transplantation following the cardiac death of donors. Previously, we showed that surfactant inhalation during warm ischemia mitigated ischemia-reperfusion injury. This study investigated the mechanisms of surfactant inhalation protection of the warm ischemic lung after reoxygenation with ventilation alone. In an isolated rat lung ventilation model, cardiac arrest was induced in the CTRL (control) and SURF (surfactant treatment) groups by ventricular fibrillation. Ventilation was restarted 110 min later; the lungs were flushed, and a heart and lung block was procured. In the SURF group, a natural bovine surfactant (Surfacten®) was inhaled for 3 min at the end of warm ischemia. In the Sham (no ischemia) group, lungs were flushed, procured, and ventilated in the same way. Afterwards, the lungs were ventilated with room air without reperfusion for 60 min. Surfactant inhalation significantly improved dynamic compliance and airway resistance. Moreover, surfactant inhalation significantly decreased inducible nitric oxide synthase and caspase-3 transcript levels, and increased those of Bcl-2 and surfactant protein-C. Immunohistochemically, lungs in the SURF group showed weaker staining for 8-hydroxy-2′-deoxyguanosine, inducible nitric oxide synthase, and apoptosis, and stronger staining for Bcl-2 and surfactant protein-C. Our results indicate that surfactant inhalation in the last phase of warm ischemia mitigated the injury resulting from reoxygenation after warm ischemia. The reduction in oxidative damage and the inhibition of apoptosis might contribute to the protection of the warm ischemic lungs.

## Introduction

To deal with the shortage of donor organs in lung transplantation, donation after cardiac death (DCD) has been introduced worldwide as a potential strategy to increase the donor pool. Since lung transplantation from uncontrolled DCD donors was first successfully performed in 2000 [1], several programs have subsequently reported acceptable outcomes comparable to donation after brain death [2], [3]. However, warm ischemia inevitably occurs in DCD donors after cardiac arrest and may cause ischemia-reperfusion (I-R) injury after transplantation.

The presence of the airway has made it possible to protect graft lungs by inflation [4], [5], ventilation with oxygen [5], [6], and drug administration, even after circulation has stopped in DCD donors. Moreover, we previously reported the protective effects of preprocurement ventilation [7] and the inhalation of several drugs [8]–[10].

A pulmonary surfactant is primarily composed of phospholipids and surfactant proteins, and functions to reduce the surface tension in the alveoli through forming a monolayer at the air-lipid interface. This mechanism prevents alveolar collapse [11], [12] and protects the lung. Surfactants have been administered in clinical settings to patients with severe primary graft dysfunction after transplantation [13], [14] and for donor lungs [15]. However, surfactant function has been reported to deteriorate with increasing warm ischemic time intervals [12]. Recently, we reported that surfactant inhalation in the last phase of warm ischemia attenuated I-R injury in an *ex vivo* rat lung perfusion model [16] and in a canine lung transplantation model [17]. We found that prerecovery surfactant inhalation improved graft lung function, maintained adenine nucleotide levels, and prevented cytokine production, resulting in attenuation of warm I-R injury [16]. Van Putte et al. also reported that exogenous surfactant resulted in anti-inflammation, which was accompanied by decreased apoptosis [18]. Hence, surfactant inhalation could serve as a potential method to reduce the severity of I-R injury. However, both reports demonstrated that various factors could have potentially induced I-R injury.

I-R commonly corresponds to anoxia-reoxygenation in organ transplantation [19]. However, the I-R injury in lungs is physiologically different from that in other organs with systemic circulation. First, the lung cells are able to maintain aerobic metabolism by using the oxygen in the alveoli even after the cessation of circulation [4], [5]. Second, the lungs can undergo reoxygenation without reperfusion, if only with ventilation. Consequently, the lung affords a unique opportunity to separate the effects of reperfusion and reoxygenation on tissue function.

We hypothesized that surfactant inhalation could mitigate hypoxia-reoxygenation injury. Here, the study aim was to clarify the effect of surfactant inhalation against injury resulting from reoxygenation with ventilation alone after warm ischemia to explore the mechanism of surfactant against I-R injury.

## Materials and Methods

### Ethics Statement

Specific pathogen-free inbred male Lewis rats weighing 285–305 g (Japan SLC, Hamamatsu, Japan) were used in these studies. All animals received humane care in compliance with the Principles of Laboratory Animal Care formulated by the National Society for Medical Research, and the “Guide for the Care and Use of Laboratory Animals,” prepared by the Institute of Laboratory Animal Resources and published by the National Institute of Health (NIH Publication 85–23, revised 1996). The study protocol was approved by the Ethical Committee of the Graduate School of Medicine at Kyoto University, Japan.

### Surfactant and Aerosol Delivery

A 30-mg/ml surfactant solution was obtained by dissolving beractant (Surfacten®, Mitsubishi Tanabe Pharma Corporation, Osaka, Japan) with normal saline (0.9%) and then aerosolized by a nebulizer (AGAL1000, Aerogen, Ireland), which was put into the inspiratory loop of the ventilator. In this system, the diameters of ∼90% and ∼60% of the aerosolized particles were maintained below 10 µm and 3.0 µm, respectively. In each experiment for the SURF (surfactant treatment) group, 0.4 mL of fluid was loaded and aerosolized for 3 min.

### Study Design

Rats were anesthetized with an intraperitoneal injection of sodium pentobarbital (50 mg/kg), intubated after tracheotomy, and ventilated with ambient air at positive pressure. Isolated rat lung ventilation was performed using a Hugo-Sachs Elektronik-Harvard Apparatus (Model 829; March-Hugstetten, Germany), as previously reported [16]. The animals were randomly allocated to three groups: Sham, CTRL, and SURF. In the CTRL and SURF groups, after a medial abdominal incision and a median sternotomy were performed, cardiac arrest was induced by ventricular fibrillation. The ventricular fibrillation was induced by a fibrillator attached directly to the right atrium and apex of the heart and continued for 7 min at 2.00 V. Cardiac arrest was defined as the complete immobility of the ventricles. After confirmation of cardiac arrest, the ventilator was stopped, and the lungs were completely collapsed. The chest was closed with skin staplers and then placed in a Styrofoam box. After 110 min of cardiac arrest period, the lungs were completely re-expanded below the pressure of 30 cmH_2_O, and positive pressure ventilation was restarted. The pulmonary artery was cannulated directly, and the lungs were flushed with 20 ml of 37°C low-potassium dextran solution (Perfadex®, Vitrolife, Uppsala, Sweden) at a pressure of 20 cmH_2_O, and drained through an incision at the apex of the left ventricle. The cannula of the pulmonary artery was pulled out, and the heart and lung block was subsequently procured and placed in the artificial thorax. The test lung was then ventilated with room air at negative pressure under the following conditions: respiratory rate = 60 cycles/min; peak inspiratory and expiratory chamber pressures = –8 and –4 cmH_2_O, respectively; ratio of inspiratory duration = 50%. The artificial thorax and the airway were water-jacketed to maintain the temperature at 37°C throughout the experiment. In the SURF group (n = 5), the surfactant was inhaled for 3 min just after the beginning of negative pressure ventilation. In the CTRL group (n = 5), no inhalation was performed. In the Sham group (n = 5), the lungs were flushed with Perfadex®, and thereafter procured and ventilated in the same way. At the end of the inhalation, the time was set as baseline, and the evaluation was commenced with airway resistance (cmH_2_O/ml) and dynamic compliance (ml/cmH_2_O) continuously monitored for 60 min.

### Adenine Nucleotide Levels

Pieces of the right middle and lower lobes of each lung were collected immediately after flushing the pulmonary vascular bed with warm saline (37°C) at the end of the evaluation. The adenosine triphosphate (ATP), adenosine diphosphate (ADP), and adenosine monophosphate (AMP) levels were measured by high-performance liquid chromatography, as previously described [16].

### Messenger RNA (mRNA) Expression

Left lung specimens were collected after the 60 min of evaluation. Five genes were examined by real-time reverse transcription-polymerase chain reaction as previously described [16]. Quantities of the genes of interest were calculated from corresponding standard curves and are presented here relative to the amount of glyceraldehyde-3-phosphate dehydrogenase (GAPDH).

### Caspase Activation Assay

Caspase-3/7 activities were measured using Caspase-Glo® 3/7 Assay kit (Promega, Madison, WI, USA) according to manufacturer’s instructions. Briefly, the supernatant of each specimen were seeded in 96-well plates and Caspase-Glo 3/7 reagent was added to each well in a 1∶1 ratio and incubated for 60 minutes before measuring luminescence as relative light units (RLUs) using a GloMax®-Multi Detection System (Promega, Madison, WI, USA). Caspase activity was normalized to the protein concentration using the Bradford protein assay. Caspase activity was represented as ratio to that in the Sham group.

### Pathological Evaluation

After 60 min of evaluation, 10% formalin was instilled intratracheally into the right upper and mediastinal lobes, which were subsequently embedded in paraffin and investigated by hematoxylin-eosin (H–E) staining.

### Immunohistochemical Analysis

The avidin-biotin complex method was used for immunohistochemical staining, as previously reported [20], [21]. The primary antibodies for incubation were as follows; 8-hydroxy-2′-deoxyguanosine (8-OHdG): Anti-8-OHdG monoclonal antibody N45.1 (JaICA, Shizuoka, Japan), inducible nitric oxide synthase (iNOS): Rabbit Anti-Human iNOS Polyclonal Antibody (Spring Bioscience, Pleasanton, USA), Bcl-2: Bcl-2 (C-2): sc-7382 (Santa Cruz Biotechnology, Santa Cruz, USA), SP-C: SP-C (FL 197): sc-13979 (Santa Cruz Biotechnology). Quantification of the immunohistological data was performed blindly by 2 independent investigators (J.S. and D.N.) as a mean of the ratio of 8-OHdG-, iNOS-, and Bcl-2-positive cells to total cells in 10 randomly chosen high-power fields (HPF) per section at a magnification of 400×. Because SP-C was produced by only alveolar type II cells, SP-C staining was assessed as a mean number of the SP-C-positive cells in the same manner.

### TUNEL Assay

Apoptotic cells were stained by the terminal deoxynucleotidyl transferase-mediated dUTP nick-end labeling (TUNEL) technique using an apoptosis in situ detection kit (Wako Jyunyaku, Osaka, Japan), as previously reported [7]. Apoptosis was expressed as a mean of the ratio of TUNEL-positive cells to total cells in the same manner as the immunohistochemical analysis.

### Statistical Analysis

All statistical analyses were performed using StatView 5.0 software (Abacus Concepts, Berkeley, California). All values are presented as the mean ± the standard deviation. Data were evaluated using one-way analysis of variance (ANOVA), Scheffe’s post-hoc multiple comparison test to explore differences between the groups. A p value <0.05 was considered statistically significant.

## Results

The body weights were similar between the Sham, CTRL, and SURF groups (297±4.0 g, 296±6.2 g, and 295±5.0 g, respectively). All lungs were ventilated and evaluated successfully in 60 min.

### Physiological Parameters

**Figure 1:**
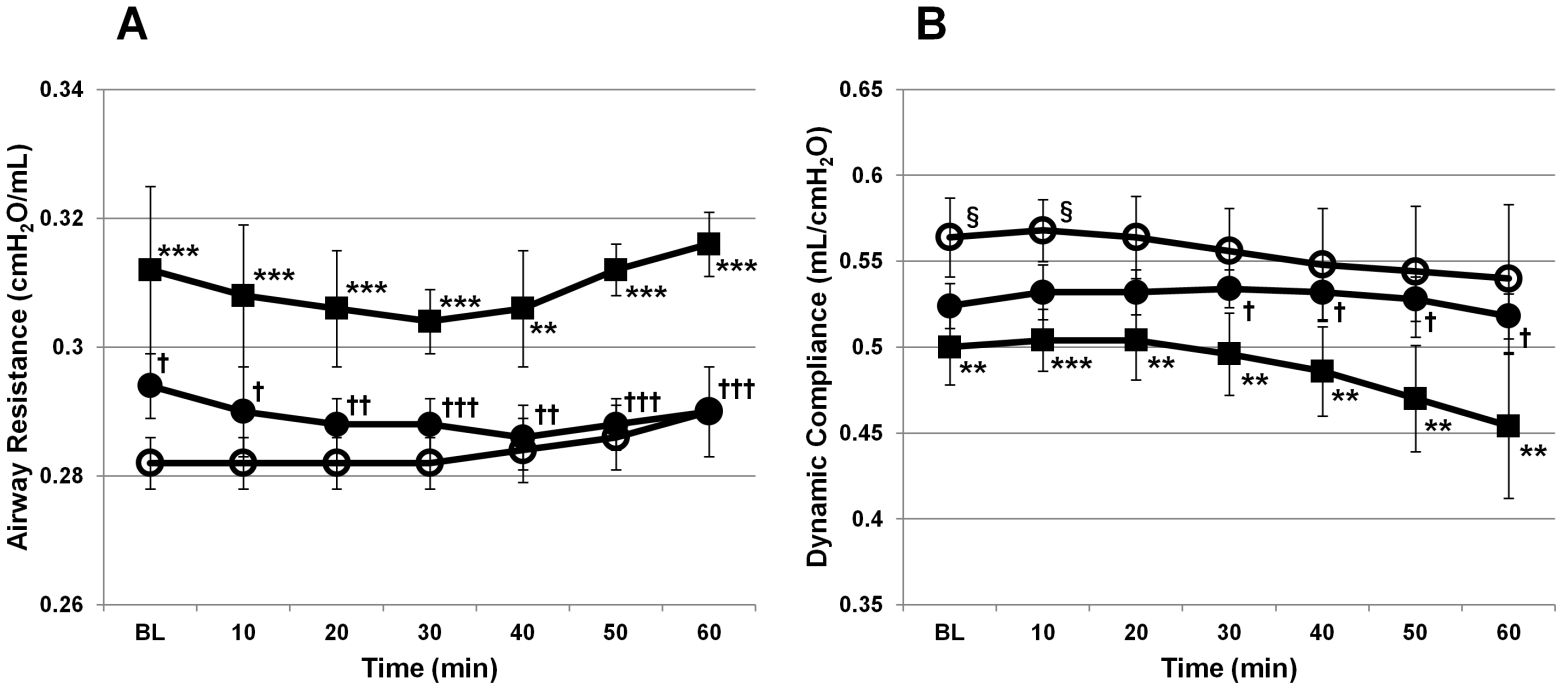
Physiological lung functions during ventilation. (A) Airway resistance. (B) Dynamic compliance. ^**^p<0.01 or ^***^p<0.001 between the Sham (open circles) and CTRL groups (boxes). ^†^p<0.05 or ^††^p<0.01 or ^†††^p<0.001 between the CTRL and SURF groups (solid circles). ^§^p<0.05 between the Sham and SURF groups. Data are expressed as mean values ± SD (BL = baseline).

In the CTRL group, airway resistance increased and dynamic compliance decreased during the latter half of the evaluation period, and were significantly higher and lower than those in the Sham group (p<0.001 and p<0.01 at 60 minutes after evaluation period). However, in the SURF group, the airway resistance and dynamic compliance were maintained at almost the same levels as those at the initiation of the evaluation, and were significantly lower and higher, respectively, than those in the CTRL group (p<0.001 and p<0.05 at 60 minutes, respectively; Figure 1A and B).

### Adenine Nucleotide Levels

**Figure 2:**
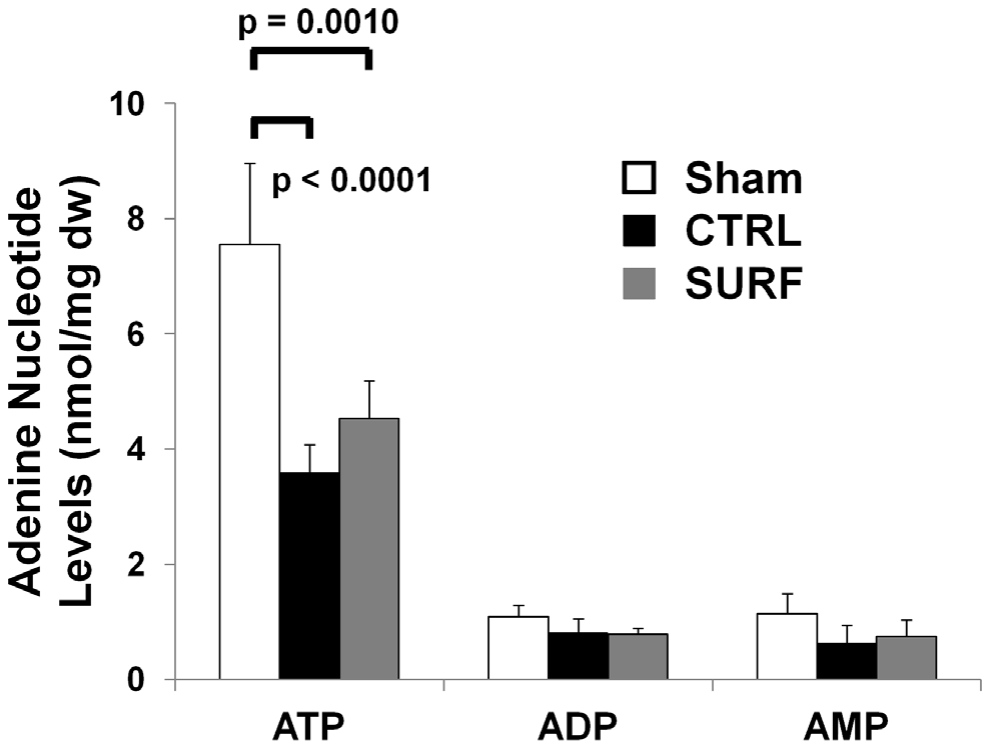
Adenine nucleotide levels. ATP, adenosine triphosphate; ADP, adenosine diphosphate; AMP, adenosine monophosphate; dw, dry weight. Data are shown as mean ± SD.

After 60 min of ventilation, although we did not have statistical significance, the ATP levels in the SURF group tended to be higher than that in the CTRL group. The differences in ADP and AMP levels between the groups did not reach significance (Figure 2).

### Evaluation of mRNA Expression

**Figure 3:**
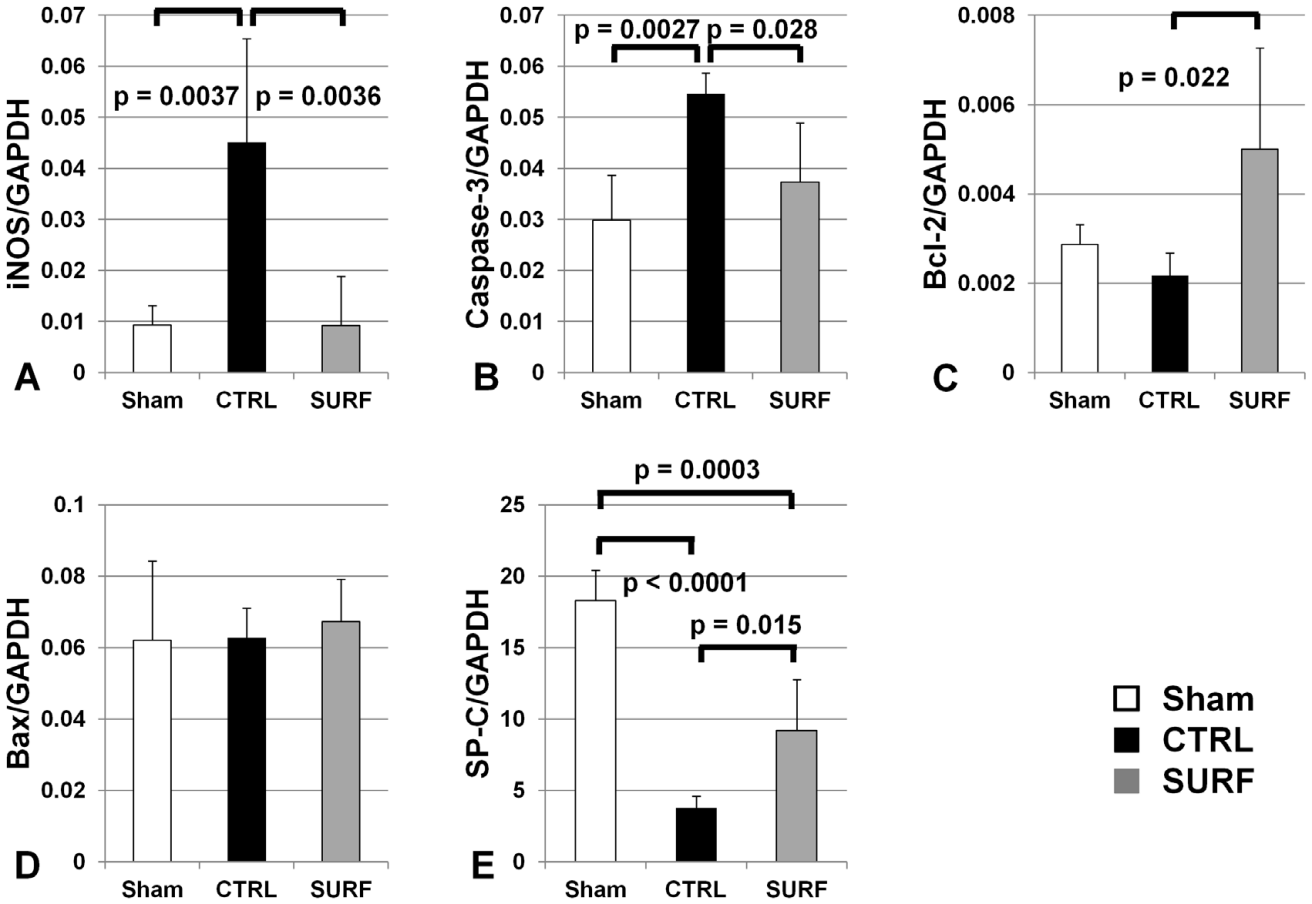
mRNA expression of cytokines after reperfusion. (A) iNOS mRNA levels. (B) Caspase-3 mRNA levels. (C) Bcl-2 mRNA levels. (D) Bax mRNA levels. (E) SP-C mRNA levels. All values are expressed as the mean ± SD.

After 60 min of ventilation, the mRNA expression of iNOS and caspase-3 was significantly lower in the Sham and SURF groups than that in the CTRL group (Figure 3A and B). Meanwhile, the B-cell lymphoma-2 gene product (Bcl-2) mRNA levels in the SURF group were significantly higher than those in the CTRL group (Figure 3C). Bcl-2 associated X-protein (Bax) mRNA levels did not differ between the study groups (Figure 3D). The SP-C mRNA levels in the SURF group were significantly lower than that in the Sham group and higher than that in the CTRL group (Figure 3E).

### Caspase Activation Assay

**Figure 4:**
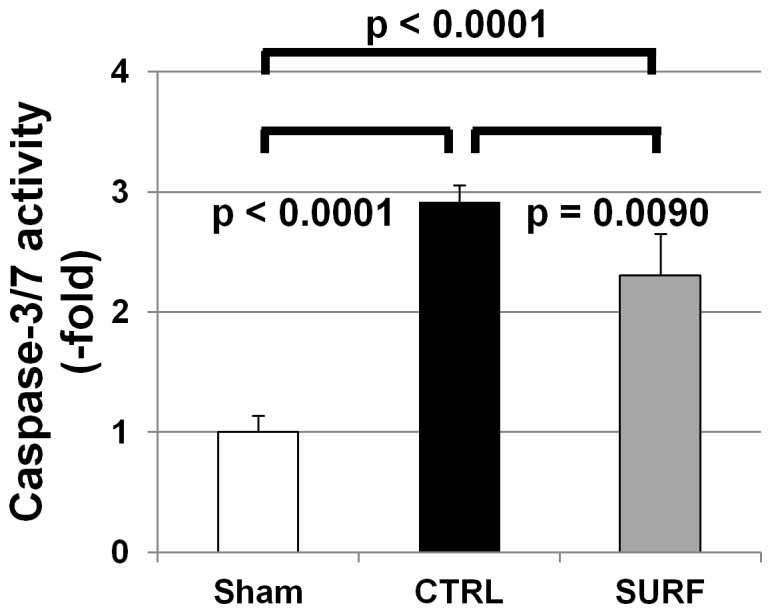
Caspase Activation Assay. All values are expressed as the mean ± SD.

After 60 min of ventilation, there was a threefold increase in Caspase-3/7 activity in the CTRL group compared to that in the Sham group. Caspase-3/7 activity in the SURF group was more than twice as much as that in the Sham group, but was significantly lower than that in the CTRL group (Figure 4).

### Pathological Examination

The lungs of 3 groups had a relatively normal structure, with no evident differences between the groups.

### Oxidative Damage After Reoxygenation

**Figure 5:**
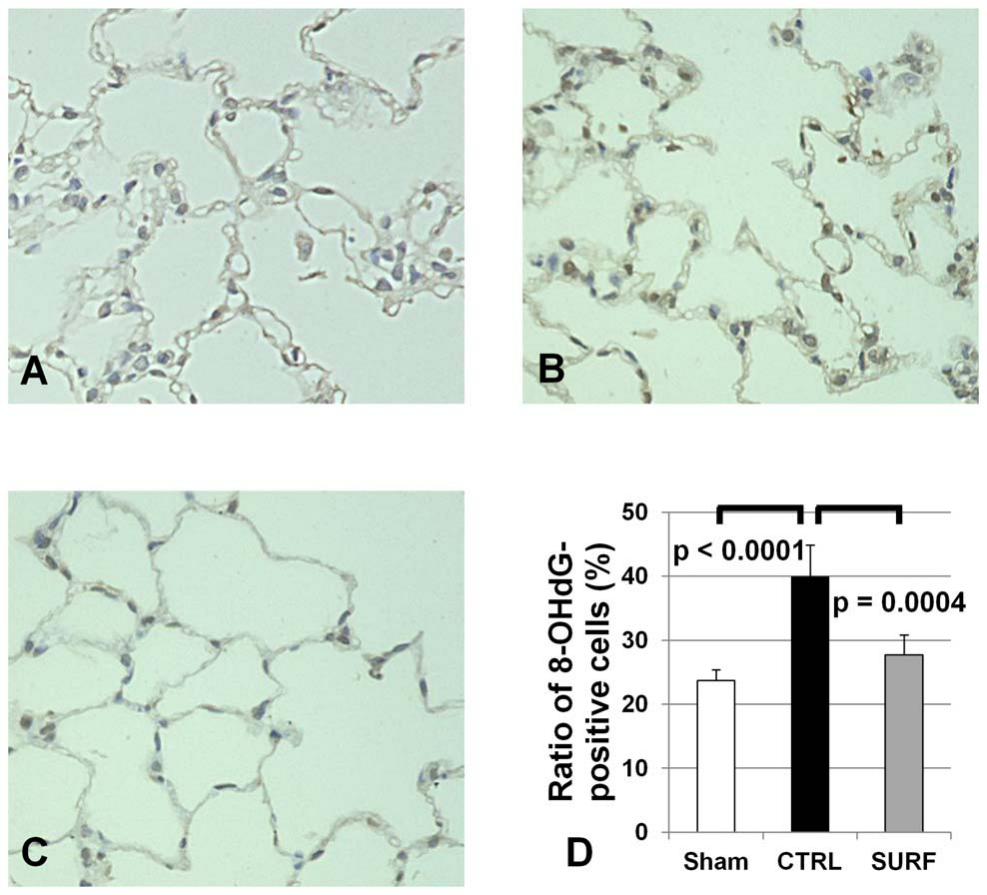
Immunohistochemical staining and the assessment for oxidative damage (original magnification 400 ×). (A) Sham group. (B) CTRL group. (C) SURF group. In the 8-OHdG staining, the ratio of 8-OHdG-positive cells was significantly lower in the Sham and SURF groups than that in the CTRL group (D). All values are expressed as the mean ± SD.

Oxidative damage was evaluated immunohistochemically by examining 8-OHdG expression [22]. The CTRL group (Figure 5B) showed more intense nuclear staining for 8-OHdG than the Sham and SURF groups (Figure 5A and C). Moreover, the ratio of 8-OHdG-positive cells was significantly lower in the Sham and SURF groups than that in the CTRL group (Figure 5D).

### Immunohistochemical Staining for iNOS

**Figure 6:**
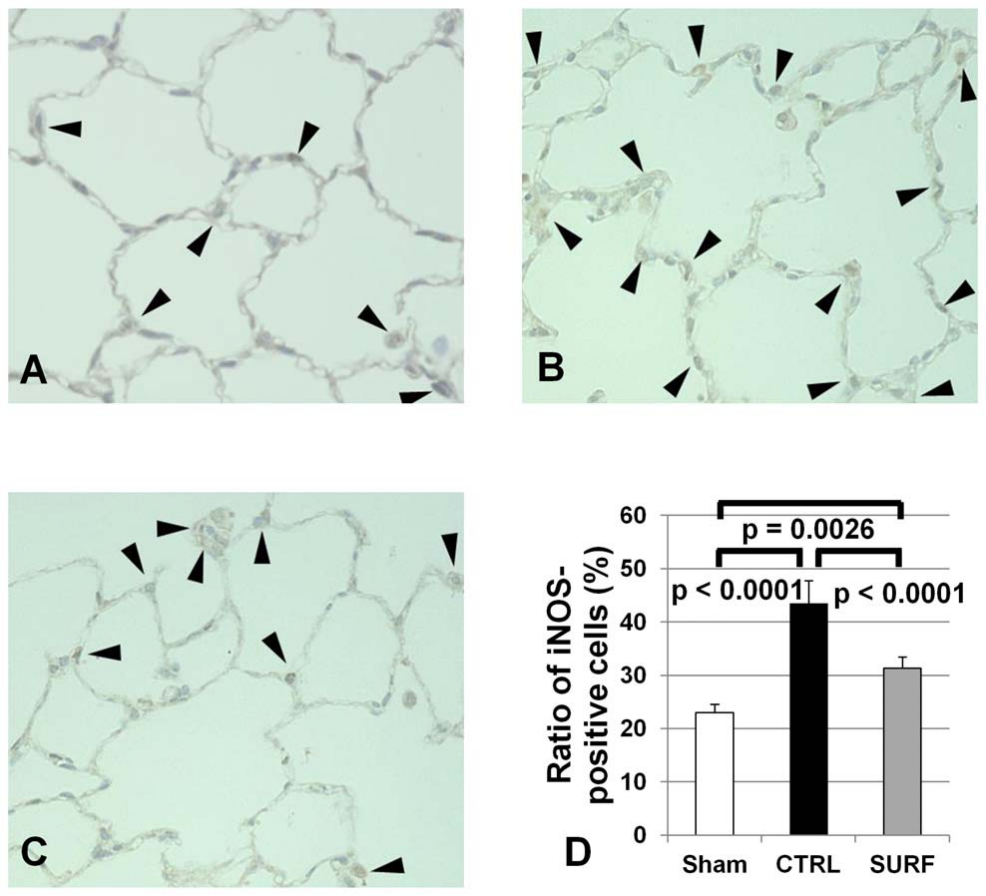
Immunohistochemical staining for iNOS (original magnification 400 ×). (A) Sham group. (B) CTRL group. (C) SURF group. Arrows indicate iNOS positive cells. The ratio of iNOS-positive cells to total cells was significantly lower in the SURF group than in the CTRL group (D). All values are expressed as the mean ± SD.

In all groups, the staining for iNOS was weak and unclear (Figure 6A, B, and C). However, the ratio of iNOS-positive cells was significantly lower in the SURF group than that in the CTRL group (Figure 6D).

### TUNEL Assay

**Figure 7:**
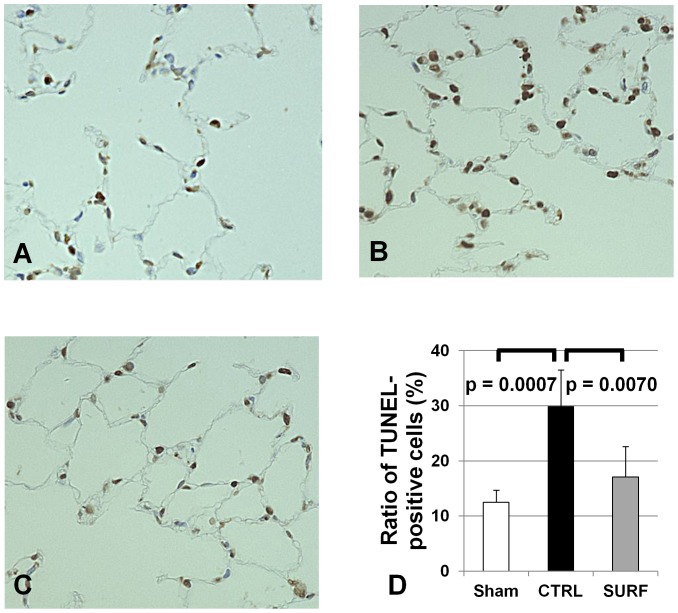
TUNEL assay (original magnification 400 ×). (A) Sham group. (B) CTRL group. (C) SURF group. The ratio of apoptotic cells to total cells in the Sham and SURF groups was significantly lower than that in the CTRL group (D). All values are expressed as the mean ± SD.

The ratio of apoptotic cells to total cells in the Sham and SURF groups was significantly lower than that in the CTRL group (Figure 7A, B, C, and D).

### Immunohistochemical Staining for Bcl-2

**Figure 8:**
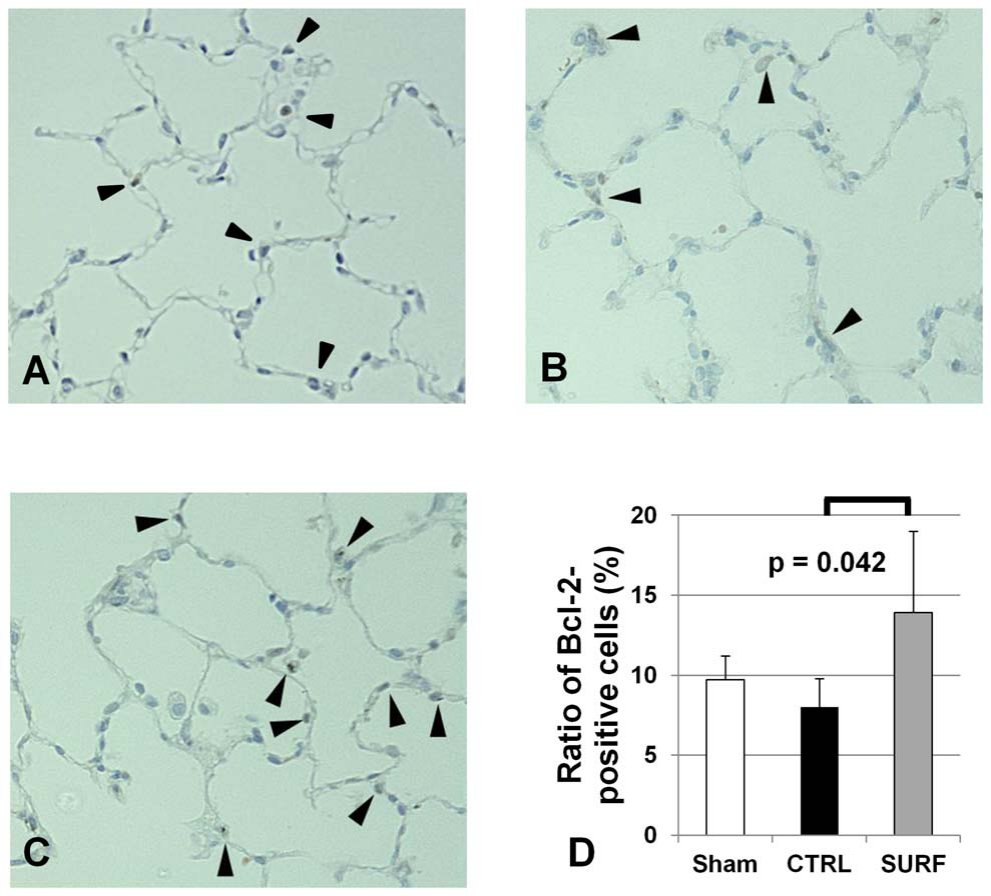
Immunohistochemical staining for Bcl-2 (original magnification 400 ×). (A) Sham group. (B) CTRL group. (C) SURF group. Arrows indicate Bcl-2 positive cells. The ratio of Bcl-2-positive cells was significantly higher in the SURF group than that in the CTRL group (D). All values are expressed as the mean ± SD.

The ratio of Bcl-2-positive cells was significantly higher in the SURF group than that in the CTRL group (Figure 8A, B, C, and D).

### Immunohistochemical Staining for SP-C

**Figure 9:**
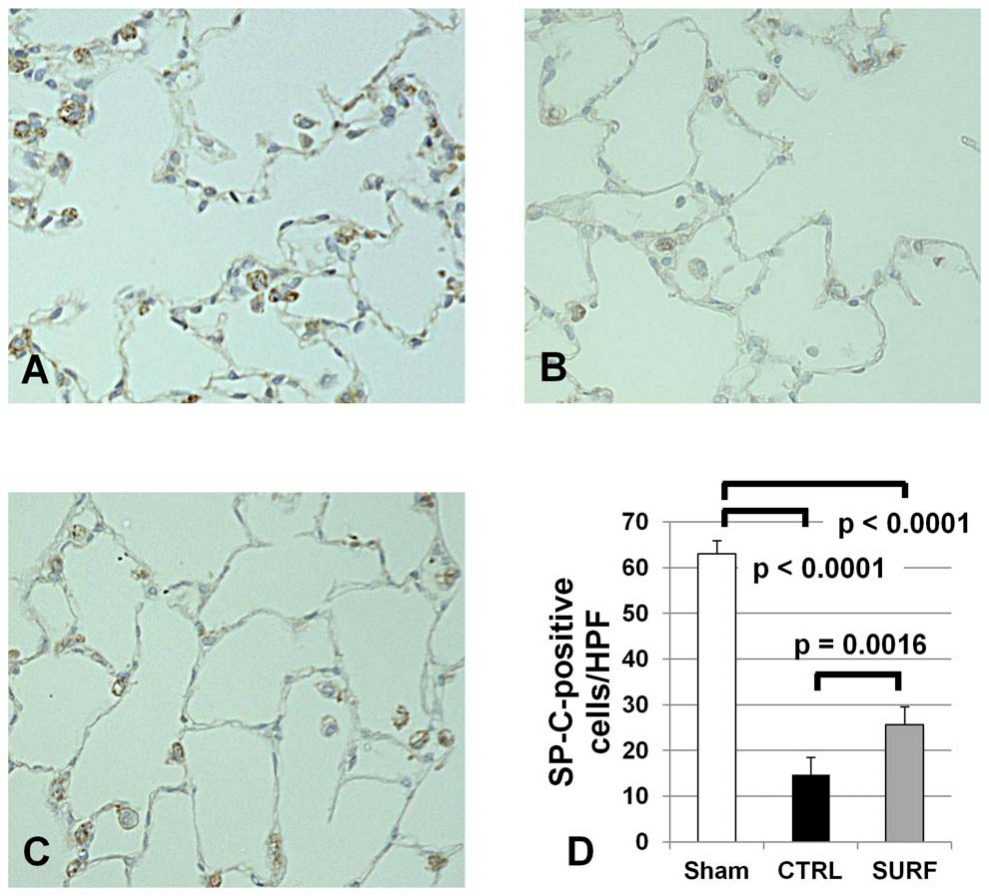
Immunohistochemical staining for SP-C (original magnification 400×). (A) Sham group. (B) CTRL group. (C) SURF group. The amount of SP-C-positive cells in the CTRL and SURF groups was significantly lower than that in the Sham group, however, that in the SURF group was significantly higher than that in the CTRL group (D). All values are expressed as the mean ± SD.

The staining of type II cells was sporadic in the CTRL group (Figure 9B), but homogeneous and clear in the Sham and SURF groups (Figure 9A and C). The amount of SP-C-positive cells in the CTRL and SURF groups was significantly lower than that in the Sham group, however, that in the SURF group was significantly higher than that in the CTRL group (Figure 9D).

## Discussion

In this study, we investigated whether surfactant inhalation in the last phase of warm ischemia could mitigate injury resulting from reoxygenation in an isolated rat lung ventilation model. We found that a reduction in oxidative stress and inhibition of apoptosis may contribute to maintaining the viability of alveolar type II cells, leading to the protection of warm ischemic lungs.

Initially, we explored the anti-oxidative effects of surfactant inhalation after resuming only the ventilation. Nitric oxide (NO) is a vasodilator and bronchodilator molecule with a critical role in numerous physiologic and inflammatory processes in the lung [23], and iNOS is present in various cell types and increased by a variety of inflammatory stimuli [24]. Large amounts of NO generated by iNOS can be toxic and pro-inflammatory [25], and may promote peroxynitrite radical formation [26]. In the current study, surfactant inhalation significantly decreased iNOS production, resulting in the reduction of oxidative damage. Van Putte et al. reported that surfactant treatment decreased iNOS expression at 30 min of reperfusion in a rat lung ischemia-reperfusion model, consistent with our results [18]. Moreover, Miles et al. showed that lung surfactant inhibited lipopolysaccharide (LPS)-induced NO production by alveolar macrophages, that the effect is due to a reduction in iNOS protein levels, and that SP-B was the surfactant component responsible for the reduction [27]. Based on these findings, we believe that surfactant prevents iNOS production, leading to a decrease in the down-regulation of surfactant by iNOS. Consistent with this theory, in the current study, the SURF group maintained predominance in the dynamic compliance.

Increased lung apoptosis has been shown to be one of the mechanisms through which iNOS and inducible NO cause I-R lung injury [28]. When NO is supplied in excess, as in hypoxia-reoxygenation situations, the part of it released from NOS can rapidly react with superoxidase to form peroxynitrite, an oxidizing agent [29]. This species can cause lipid peroxidation, apoptosis, alterations in DNA, and protein nitration and oxidation, all of which can lead to profound cellular disturbances [30]. Therefore, based on our data demonstrating that surfactant reduced oxidative damage, we hypothesized that surfactant may induce apoptotic proteins. We used assays for activated caspase-3 activity, a terminal effector of apoptosis [31], and found that surfactant inhalation significantly reduced caspase-3 mRNA levels and caspase-3/7 activity, consistent with van Putte et al. [18]. This result could explain the reduced apoptotic cells in the SURF group. Bcl-2 is a well-studied antiapoptotic protein, and is thought to inhibit apoptosis by stabilizing the outer mitochondrial membrane, preventing release of cytochrome *c* into the cytosol and thereby blocking caspase activation [32]. In the current study, mRNA and protein expressions of Bcl-2 in the SURF group were significantly higher than those in the CTRL group. Prior studies have demonstrated that Bcl-2 overexpression in donor grafts is cytoprotective [31], [33], [34]. Further, caspase-3-dependent cleavage of Bcl-2 promotes further caspase activation as part of a positive feedback loop for executing the cell [35]. Reduced caspase-3 activity by surfactant inhalation might cut off the feedback loop for apoptosis.

Mitochondrial membrane permeability is directly controlled by the Bcl-2 family of proteins primarily through regulating the formation of apoptotic protein-conducting pores in the outer mitochondrial membrane [36]. The inhibition of apoptosis could lead to the maintenance of cell viability. Furthermore, ATP synthesis, coupled to cellular respiration, occurs in the mitochondria, and irreversible damage at the level of oxidative phosphorylation can lead to cell death by energy deprivation [37]. The current study showed that surfactant inhalation led not only to the inhibition of apoptosis but to the maintenance of ATP levels. In addition, the levels of SP-C, a marker of cell viability of type II cells, were also maintained more in the SURF group than in the CTRL group.

Our study had several limitations. First, a small animal *ex vivo* model was used as opposed to an actual lung transplantation model. Second, no cold preservation was applied, which is always required clinically; and the lungs were ventilated with negative pressure. Third, the lungs were completely collapsed during warm ischemia, which is different from clinical settings.

In conclusion, we confirmed that only 3 min of surfactant inhalation in the last phase of warm ischemia mitigated the injury resulting from reoxygenation in an isolated rat lung ventilation model. The reduction in oxidative damage and the inhibition of apoptosis may contribute to the protection of the warm ischemic lungs in our study. Hence, surfactant inhalation could be a convenient and effective method for improving graft function in lung transplantation from DCD donors.
